# Adaptation and validation of the Spanish version of the everyday cognition battery for assessing everyday cognition in older adults

**DOI:** 10.1186/s12877-022-02944-5

**Published:** 2022-03-22

**Authors:** Raquel Jimenez Gomez, Eduardo Jose Fernandez Rodriguez, Celia Sanchez Gomez, Juan Jesus Cruz Hernandez, Maria Isabel Rihuete Galve

**Affiliations:** 1 University of Salamanca, Salamanca, Spain; 2 Salamanca Biomedical Research Institute (IBSAL), Salamanca, Spain; 3 Salamanca University Assistance Hospital Complex, Salamanca, Spain

**Keywords:** Cognitive impairment, Everyday cognition, Everyday cognition battery

## Abstract

**Introduction:**

Ageing entails a series of neuroanatomical and neurophysiological changes in some cognitive processes that directly affect the daily life and autonomy of a person. We believe it is necessary to have tools that assess the cognitive functions that are essential for carrying out daily activities in an independent manner. The aim of this study was to translate the Everyday Cognition Battery (ECB) into Spanish, adapt it to the sociocultural context of Spain, and validate it by testing the psychometric properties, i.e., the reliability and validity of the translated version.

**Methods:**

The translation and adaptation of the ECB into Spanish was carried out following the method recommended by Beaton et al., the process concluding with a pilot test to ensure that subjects were able to understand the scale correctly. Between March and October 2019, the study population voluntarily completed the translated version of each of the four subscales that make up the battery of tests.

The translated version was validated by analysing its psychometric properties, using reliability or internal consistency tests assessed with Cronbach’s alpha coefficient and validity tests analysed using correlation tables and Spearman’s correlation coefficient. The scale considered to represent the gold standard in the assessment of cognition was the Rapid Assessment of Cognitive Functions (RACF), and to assess Instrumental Activities of Daily Living (IADL) this was the Lawton and Brody Index.

**Results:**

The total study population included 226 subjects, of which 52 participants were excluded, resulting in a study sample size of 174 older adults. The recognition, inductive reasoning and computation span tests showed good reliability (Cronbach’s alpha coefficient of > 0.827, > 0.836, and > 0.823, respectively), while the knowledge test showed questionable reliability with a Cronbach’s alpha coefficient of > 0.615. The validity analysis demonstrated that all the combinations of correlations of the different scales were significantly and positively related to one another.

**Conclusions:**

The Spanish version of the ECB tool is socially and culturally equivalent to the original version, and both its validity and reliability for assessing everyday cognition in older adults have been demonstrated.

## Introduction

Today’s population is undergoing a demographic shift towards a longer-lived society. The progressive ageing associated with an increased life expectancy and a decreased fertility rate is one of the greatest challenges facing our society [1, 2]. All developed societies are now characterised by a progressive ageing of the population and Spain is no exception to this trend. According to data from the United Nations (2017), it is estimated that by 2050, Spain will be the second most aged country in the world, with 41.9% of the population being 60 years of age or older [3].

Ageing entails a series of neuroanatomical and neurophysiological changes that manifest as alterations in cognitive functions, involving a slower information processing speed and deficits in psychomotor skills and perceptual abilities that directly affect a person’s daily life and autonomy. Cognition is the ability of human beings to gather, organise, assimilate, process and use information on their environment, which they receive through a variety of cognitive pathways and processes, converting it into knowledge that enables them to interpret the world around them, adapt to it, and function in their surroundings [4, 5]. Given the important implications of daily cognition for everyday life, there is growing interest in this area of research [6, 7]. As early as 1996, authors such as Willis et al. defined the concept of everyday cognition as the ability of human beings to adequately perform the complex cognitive tasks of day-to-day life, which are considered essential for living autonomously in society [8]. Several authors [6, 9] consider cognition and intelligence to be adaptive functions necessary for the survival of the individual and suggest the need for specific tools to assess cognitive functions in the everyday world [8, 10].

The impact of cognitive loss on everyday function is a major concern for adults and one of the most feared aspects of ageing, together with the loss of mental faculties and autonomy, so early detection and systematic characterisation of cognitive and functional loss has important clinical and scientific applications [11]. Due to the impact the loss of cognitive function has on older adults and the high costs involved in the multidisciplinary treatment of neurodegenerative diseases, the study of cognitive function in older adults in everyday life is currently on the increase [12, 13].

Nowadays, the use of scales has become widespread with the aim of ascertaining the patient’s baseline status, determining the impact of the current disease, transmitting objective information, monitoring changes and, ultimately, establishing specific treatments and assessing the patient’s response to these; all of which favours the unification of assessment criteria for individuals as well as with other professionals, increasing the objectivity of the information we transmit and boosting clinical research [14].

In recent decades, several measurement instruments have been developed to assess cognitive ageing [8, 10, 15]. These instruments have focused on Activities of Daily Living (ADL) (medication use, financial planning, meal preparation, family management), domains considered essential for independent living [10, 16, 17]. One such tool is the Everyday Cognition Battery (ECB) [10, 11], used to assess daily living skills in older adults with no cognitive impairment. The ECB was developed in 1999 by Allaire JC and Marsiske M [10, 16]. from Wayne State University in Detroit, Michigan, to assess cognitive competence in three instrumental domains of daily living through the performance of important everyday instrumental tasks: medication use, financial planning and management, and nutrition and food preparation. These instrumental tasks have been described as universal, basic and mandatory [7] and are considered necessary for older adults to be able to function independently in the real world [18], and can be considered highly predictive indicators of institutionalisation [18, 19] and mortality in older adults [20, 21]. The ECB comprises four tests, each designed to assess a single cognitive ability: Knowledge Test (subscale with a maximum score of 30 points), Inductive Reasoning Test (with a maximum score of 84 points), Recognition or Declarative Memory Test (with a maximum score of 30 points) and Computation Span Test (subscale in which a maximum score of 56 points can be obtained). The total score of the ECB is 200 points [22].

Taking these aspects into account, together with the fact that in Spain we have no validated instrument for assessing everyday cognition in older adults, the aim of this study was to translate, adapt and validate the ECB, a tool for assessing everyday cognition in older adults with no previously diagnosed cognitive impairment, into Spanish.

## Method

### Study design and participant selection

This is a descriptive cross-sectional study that included 226 participants, healthy older adults from the town of Salamanca, over 60 years of age, who participated in the Occupational Therapy Programmes, carried out at the University of Salamanca with the collaboration of the Faculty of Nursing and Physiotherapy, through an agreement with the city council’s Department for the Elderly and various associations.

The sample was selected by means of a random cluster sampling of 9 associations in the city and, from each of these, the participants were randomly selected from the set of people who were willing to complete the study. The sample size decision was calculated considering the recommendations of Carretero H [23], we also considered the sample size used by the authors in the development of the everyday cognition battery, which was the same as the one we used in our adaptation and validation in Spanish (*n* = 226).

In order to be included and be able to participate in the study, the participants had to meet the following inclusion criteria: be of either sex, over 60 years of age, not present cognitive impairment (score less than 46 points on RACF scale), participate in the Occupational Therapy programmes, and voluntarily accept their inclusion in the study by signing the informed consent form. The exclusion criteria were: not being able to read or write and having severe hearing loss or blindness. The following withdrawal criteria were also considered after inclusion: abandonment of the study in any of its phases despite having signed the informed consent form, and completion of less than 50% of the scales administered.

The variables included in the study were sociodemographic and health characteristics and the assessment instruments, the scale to be validated, ECB, and the comparison scales considered to be the gold standard for cognitive assessment, the Rapid Assessment of Cognitive Function (RACF) [24], and the Instrumental Activities of Daily Living (IADL), Lawton and Brody scale [25]. The choice of these scales is based on the dual evaluative capacity of the ECB, on the one hand, the evaluation of cognitive function, hence the lesson of the RACF; and on the other hand, the evaluation of these cognitive functions during the performance of activities of daily living, or the resolution of everyday cognitive problems, with particular emphasis on the performance of instrumental activities of daily living. This is why we chose, secondly, the Lawton and Brody scale.

All data collected through the participant assessments was gathered by the same person, the principal investigator.

### Translation of the everyday cognition battery assessment scale into Spanish

The translation [26, 27] of the ECB scale into Spanish was carried out following the method recommended by Beaton et al. [28], in the following stages: translation from the original language (English) into Spanish, by two independent translators; synthesis and comparison of the two translated versions; back-translation or reverse translation into the original language of the scale; evaluation of the two translations by a research group or committee (made up of two doctors from the health field with doctorates from the University of Salamanca, an external researcher specialising in cognitive impairment and the principal researcher) with the aim of resolving any discrepancies and consolidating a pre-final version of the instrument to be used in the final-stage pilot test, to be administered to a reduced sample of participants with similar characteristics to the target population in order to check the adaptation and understandability of the ECB translated into Spanish, to identify possible shortcomings and to rectify any errors.

### Questionnaires

The sociodemographic characteristics assessed in the study were age; sex; marital status (single, married, widowed or divorced); level of education according to the International Standard Classification of Education 2011 (none, primary, secondary or higher); living arrangements, whether they live alone or not; physical exercise habits, whether they do physical exercise or not and the days per week they exercise; and drug treatment only including the two large pharmacological groups that can modify the cognitive level of patients, benzodiazepines and opiates. All these sociodemographic characteristics were recorded in a document entitled Clinical History.

The assessment scales used in the study were, apart from the ECB scale, those considered to be the gold standard for assessing cognitive function and IADL.

The RACF [24] is a neuropsychological test that assesses cognitive ability and allows a rapid assessment of possible cognitive impairment. This scale is validated for the Spanish population.

After analysis of the results obtained in the pilot test, the inclusion of an IADL assessment scale in the study was positively assessed, the scale selected being the Lawton and Brody Index. This scale assesses the activities considered necessary for individuals to be able to maintain themselves independently at home and remain independent in the community; it is also validated for the Spanish population.

### Statistical analysis

The statistical analysis included a descriptive analysis of the sociodemographic characteristics of the sample and the scores from each of the assessment instruments used, as well as an analysis of the psychometric properties of the Spanish version of the ECB.

For the descriptive analysis we used the mean, standard deviation, minimum and maximum for quantitative variables, and counts and percentages for qualitative variables.

To ascertain the psychometric properties of the Spanish version of the ECB, various tests were used to assess the validity and reliability of the instrument.

Reliability: as the questionnaire comprises several subscales, each of which measures a different dimension of the same phenomenon, the reliability of each of the subscales has been assessed. Reliability has been assessed by means of internal consistency, the most commonly used statistic being Cronbach’s alpha coefficient [29–31]. To interpret Cronbach’s alpha coefficient values, we followed the recommendations of George Mallery (2003), [32].

Validity: the validity analysis was carried out using correlation tables, the statistical resource used being Spearman’s correlation coefficient -Spearman’s rho- which presents values between − 1 and 1 [30, 33]. Correlations were made between the Spanish version of the total ECB and its four subscales with the scale considered as the gold standard for cognition in the study, the RACF, and with the IADL assessment scale, the Lawton and Brody Index.

The Statistical Package for Social Science (SPSS), version 26 (IBM Corp., 2019) was used to analyse the data.

### Ethical and legal aspects

This study complies with the requirements of the Ethics Committee of the University of Salamanca and the Code of Ethics of the World Medical Association (Declaration of Helsinki). Written informed consent was obtained from all subjects. All the information on the results has been processed and stored in a strictly confidential and anonymous manner in accordance with the provisions of Organic Law 15/1999 of 13 December on the Protection of Personal Data. The study was approved by the Bioethics Committee of the University of Salamanca on 31 October, 2018. The project was also approved and included in the Clinical Trials Register (Clinical Trials. Gov. PRS) with registration number: NCT 04169633.

## Results

### Translation of the ECB

Following the recommendations of Beaton et al. [28], two translations were made from the original English into the target language, Spanish; one translation was done by the Central Language Service of the University of Salamanca, and the other by the principal researcher. The research group carried out an analysis and comparison of the two translations and evaluated and corrected the discrepancies found. The items that most hindered comprehension were specific foods that in Spain, due to cultural customs, are not commonly consumed, as well as the name of a medicine that in Spain is either unknown or referred to by a different name. All opinions and discrepancies were compiled by the research group and, through consensus and prior justification, the items were replaced by similar items encountered in the target culture. In this way, the first Spanish version of the ECB was obtained so that it could be used in the pilot test.

The pilot test was carried out with 14 participants, who showed no comprehension difficulties in terms of the questions and items constituting the first Spanish version of the ECB, so no further adjustments were made to it.

### Descriptive statistics: characteristics of the participants

Following the pilot study, the total study population contacted by the researcher was 226 subjects, all healthy older adults with no cognitive impairment. 52 participants were excluded because they did not meet the inclusion criteria, or met the exclusion criteria or some withdrawal criteria; most decided to withdraw from the study, despite having signed the informed consent form, due to the length of time required to complete the battery.

The final sample size of the study was therefore 174 participants (*n* = 174). The mean age of the study participants was 73.3 years (± 7.11 years); 73.6% were women; 6.9% were single; 54% were married; 33.9% were widowed; and 5.2% were divorced. In terms of the educational level of the participants, 6.9% had no education at all, 50.6% were educated to primary school level, 19.5% to secondary school level, and 23% to higher education level. 32.8% of the study participants habitually lived alone. 86.2% of the study subjects exercised during the week and the average number of days they exercised was almost 3 days a week (2.91 days). Of the two groups of medications considered in the study, none of the study participants were taking opiates long-term, and only 15.5% were taking some form of benzodiazepine on a regular basis (Table 1).

**Table 1:** Characteristics of study participants: sociodemographic variables

	Participants (***N*** = 174)
**Age** (years)	73.30 (±7.11)
**Sex**	
Male	46 (26.4%)
Female	128 (73.6%)
**Marital status**	
Single	12 (6.9%)
Married	94 (54%)
Widowed	59 (33.9%)
Divorced	9 (5.2%)
**Educational level (ISCED, 2011)**	
No education	12 (6.9%)
Primary Ed.	88 (50.6%)
Secondary Ed.	34 (19,5%)
Higher Ed.	40 (23%)
**Living arrangements. Do you live alone?**	
No	117 (67.2%)
Yes	57 (32.8%)
**Physical exercise**	
No	24 (13.8%)
Yes	150 (86.2%)
**Physical exercise days**	2.91 (±1.70)
**Medication taken**	
None	147 (84.5%)
Benzodiazepines	27 (15.5%)
Opiates	0 (0%)

The values are the mean and standard deviation for quantitative variables, and counts and percentages for qualitative variables

### Descriptive statistics

In the descriptive analysis of the results obtained in the different rating scales administered, we considered the questions or items answered correctly.

The total mean score obtained in the global everyday cognition scale was 154.27 points (SD = 19.28) out of a total of 200 points. In the Knowledge Test, the mean number of questions answered correctly was 18.82 (SD = 3.76) out of a total of 30 questions; in the Recognition or Declarative Memory Test, the mean number of questions answered correctly was 23.28 (SD = 4.81) out of a total of 30 questions; in the Inductive Reasoning Test, the mean total score obtained was 61.09 points (SD = 11.44) out of a total of 84 points; and, finally, in the Computation Span Test, the mean number of questions answered correctly was 51.03 (SD = 4.97) out of a total of 56 questions.

The average total score obtained on the RACF was 51.76 out of 56 points, and the average total score obtained on the Lawton and Brody Index was 7.43 out of 8 points. (Table 2)

**Table 2:** Descriptive statistical analysis of the scales used

	Participants (***N*** = 174)
Everyday Cognition Battery	154.27 (± 19.28) / 200
Knowledge Test	18.82 (± 3.75) / 30
Recognition Test - Declarative Memory	23.28 (±4.81) / 30
Inductive Reasoning Test	61.09 (± 11.43) / 84
Computation Span Test	51.03 (± 4.96) / 56
RACF	51.67 (± 4.24) / 56
Lawton and Brody Index	7.43 (± 1.07) / 8

The values are the mean and standard deviation of the correctly answered scores for each of the scales used

### Reliability

To demonstrate the reliability of the Spanish version of the ECB, an internal consistency study was carried out, using Cronbach’s alpha coefficient as a statistical tool, and this was then compared to the results obtained in the original version of the ECB. The internal consistency estimates, using Cronbach’s alpha coefficient, in the original version of the ECB are very similar to those of the Spanish version of the ECB, indicating that each subscale of the test contains a relatively homogeneous set of items.

According to the recommendations of George Mallery, three of the four subscales present good reliability, with a Cronbach’s alpha coefficient above 0.8 (Recognition or declarative memory test α = 0.82, Inductive Reasoning test α = 0.83, and Computation Span test α = 0.82). The Knowledge Test presented a questionable Cronbach’s alpha coefficient of just over 0.6 (α = 0.61), similarly to the original version of the battery. (Table 3)

**Table 3:** Reliability of the Spanish version of the ECB

Reliability: internal consistency (Cronbach’s alpha coefficient)	
Knowledge Test	α = 0.61
Recognition Test - Declarative Memory	α = 0.82
Inductive Reasoning Test	α = 0.83
Computation Span Test	α = 0.82

### Validity

To analyse the validity of the Spanish version of the ECB, correlation tables were used, which were resolved using Spearman’s Correlation Coefficient (Spearman’s rho). Correlations were made between the ECB and the scale considered the gold standard for cognition assessment, the RACF, and with the IADL assessment scale, the Lawton and Brody Index.

Firstly, the total score of the Spanish version of the ECB and its four subscales was correlated with the total score obtained in the RACF, resulting in a Spearman’s rho correlation index of > 0.4 with a significance of *p* < 0.001 in all cases, indicating the existence of a significant and positive relationship between the variables analysed. Secondly, the total score of the Spanish version of the ECB and its four subscales was correlated with the total score obtained in the Lawton and Brody Index, resulting in a Spearman’s rho correlation index of > 0 with a significance of *p* < 0.001 in all cases, confirming the existence of a significant and positive relationship between the variables analysed. (Tables 4, 5)

**Table 4:** Validity of the Spanish version of the ECB

**RACF**	Total ECB	Spearman correlation (RHO): 0.602; *p* < 0.001
	Knowledge	Spearman correlation (RHO): 0.447; *p* < 0.001
	Recognition - Declarative memory	Spearman correlation (RHO): 0.485; *p* < 0.001
	Inductive Reasoning	Spearman correlation (RHO): 0.419; *p* < 0.001
	Computation Span	Spearman correlation (RHO): 0.635; *p* < 0.001

Results of correlations of the total ECB score and each of its four subscales with the total RACF score

**Table 5:** Validity of the Spanish version of the ECB

**Lawton and Brody Index**	Total ECB	Spearman correlation (RHO): 0.446; *p* < 0.001
	Knowledge	Spearman correlation (RHO): 0.207; *p* < 0.001
	Recognition - Declarative memory	Spearman correlation (RHO): 0.175; *p* < 0.001
	Inductive Reasoning	Spearman correlation (RHO): 0.281; *p* < 0.001
	Computation Span	Spearman correlation (RHO): 0.281; *p* < 0.001

Results of correlations of the total score of the ECB and each of its four subscales with the total Lawton and Brody Index score

## Discussion

As a consequence of the need to respond to a clinical and social question which has considerable repercussions in today’s society due to the progressive ageing of the population, this study was conducted to provide a tool validated for the Spanish population to measure the daily cognitive performance of older adults as a reflection of their functional and psychosocial capacity, important indicators of institutionalisation and mortality in this population group.

On a strictly theoretical level, the best way to assess older adults’ everyday cognition is by measuring competence, rather than by measuring their everyday performance. The assumptions and tasks that make up the ECB are based on familiar functional domains and specific tasks that they should be able to solve, rather than tasks that they actually perform in everyday life; that is, they are shown assumptions or tasks that adults do not encounter on a daily basis, but their ability to perform them when the need arises is of great importance as it may have important repercussions for maintaining their independence and autonomy in society.

This study is the first formal evaluation of the ECB scale in a language and population other than the one in which it was created; therefore, the principles and methods used for the creation of the original scale were followed in the development of the present study. There is a previous study by Russo et al. [34] where a similar assessment in terms of everyday cognition is presented, but we consider that there is still insufficient scientific evidence in such a broad field of future interest.

During the process of adapting the scale, it is important to highlight that, after obtaining the approval of its creators, Allaire J., the linguistic, semantic and conceptual equivalence with the original version has been maintained during translation and cross-cultural adaptation in order to maintain the appearance and content of the new version of the scale.

The applicability of instruments in countries with languages and cultures different from the country of origin in which they have been developed can be problematic. The application of a scale to subjects from a culture other than the one in which it was created entails a translation process to achieve a linguistic adaptation, and a validation process to obtain certification of the scale by establishing its psychometric properties of validity and reliability.

The results of this study are in line with those obtained by the original author of the scale in the English language, and an American culture, and, therefore, we can affirm that the validated Spanish version of the ECB meets the accepted standards of reliability and validity.

To demonstrate the reliability of the Spanish version of the ECB, we used internal consistency, the results of which allowed us to establish that the assessments of the Spanish version and the original English version are very similar. Three of the four subscales present a good reliability according to the recommendations of George Mallery, while the Knowledge Test presents questionable reliability, but in line with the original ECB scale.

To demonstrate the validity of the Spanish version of the ECB, correlation tables were used, the most important measure of which is Spearman’s correlation coefficient (Spearman’s rho). The ECB was correlated with the scale considered in this study to be the gold standard, the RACF, and the IADL assessment scale, the Lawton and Brody Index. All the correlations carried out established a significant relationship between the variables analysed. A weaker correlation was observed with the Lawton-Brody scale.

To ensure the proper comprehension of the four ECB subscales, a small modification was made to the Computation Span test, consisting of a double reading of the problems to be answered by the participants.

### Strengths and limitations

This is the first formal evaluation of the ECB scale in a language and population other than the one in which it was originally created.

The ECB is a standardised instrument that offers a series of benefits: it provides a wide range of information on a person’s everyday cognition, it contributes information on a person’s knowledge, declarative memory, recognition and calculation in terms of instrumental activities considered essential in everyday life for a person to remain autonomous and independent in society. The ECB makes it possible to predict the cognitive trajectories of everyday functional tasks of older adults, while at the same time allowing an early diagnosis of possible cognitive impairment. As a consequence, early interventions can be made to prevent the progression of this cognitive impairment, thereby reducing the burden on healthcare, economic and family services.

All the assessments were conducted by the same person, reducing possible differences in the administration of the tests and making the procedure for administering the questionnaires more rigorous and precise.

One of the strong points of the method used was the implementation of a pilot test that included the evaluation of a pre-final version with the aim of identifying shortcomings and rectifying errors.

The following limitations were also observed: the subjects were from a single provincial capital (Salamanca). Despite the advantages of sample homogeneity, it is recommended that the sample be extended to include subjects from other autonomous communities in Spain, or other Spanish-speaking countries with similar customs. The average time required to administer the complete battery is too long for it to be used as a routine tool in a standard consultation; however, subscales could be administered in separate sessions. The age range of the study participants is very wide, for which reason the results of this research may be slightly biased.

Finally, the voluntary and altruistic nature of the study may be considered a limitation in that patients who were more likely to complete the questionnaires correctly were more likely to accept inclusion in the study than those with poorer performance.

### Future research

Since research never finishes, and with the aim of improving our knowledge of cognition, new challenges lie ahead.

Given that the age range of the study sample is very wide and the current trend in defining the concept of old age is increasingly removed from the chronological perspective, as a consequence of lifestyle improvements, a possible line of research could be to assess the same concept in populations with narrower age ranges, in order to more accurately assess the impact of age on cognitive function.

Likewise, we consider that future research should involve a reduction in the proportional sex-related differences in order to more precisely assess cognitive impairment as a function of this variable, thus evaluating the impact of sex on cognitive function in older adults.

Along similar lines, it would be interesting to apply the translated Spanish version of the ECB to samples with different neuropsychiatric conditions, in order to assess everyday cognition in groups of patients with different pathologies.

## Conclusions

The Spanish version of the Everyday Cognition Battery is a validated, useful, practical, simple and standardised instrument for assessing everyday cognition in older adults with no previously diagnosed impairment.

## Data Availability

The datasets used and/or analysed during the current study available from the corresponding author on reasonable request. The scale adapted and translated into Spanish can be requested by any interested author by contacting the corresponding author.

## Abbreviations

ADL: Activities of Daily Living
ECB: Everyday Cognition Battery
RACF: Rapid Assessment of Cognitive Functions
IADL: Instrumental Activities of Daily Living
ISCED: International Standard Classification of Education
